# DNA-Immobilized Special Conformation Recognition of L-Penicillamine Using a Chiral Molecular Imprinting Technique

**DOI:** 10.3390/polym14194133

**Published:** 2022-10-02

**Authors:** Lianming Zhang, Kui Luo, Jingxia Gao, Jianping Li

**Affiliations:** College of Chemistry and Bioengineering, Guilin University of Technology, Guilin 541004, China

**Keywords:** molecularly imprint, sensor, dsDNA, chiral, L-Pen

## Abstract

A new chiral molecularly imprinted polymer (MIP) sensor with dual recognition ability was developed for the highly selective separation of enantiomers with toxic side effects in drugs. The sensor contains double-stranded deoxyribonucleic acid (dsDNA) as the element that immobilizes the chiral molecular conformation: the dsDNA enables the imprinted cavities to match the three-dimensional structure and functional groups from the chiral molecule. By embedding the spatial orientation of dsDNA in MIPs, one can accurately capture and immobilize the molecular conformation, eliminating the influence of interfering analogues. Herein, L-penicillamine (L-Pen) was selected as the chiral template molecule and embedded into dsDNA to form dsDNA-L-Pen complex, which was then embedded into the MIPs by electropolymerization. After elution, the stereo-selective imprinted cavities were obtained. The ATATATATATAT-TATATATATATA base sequence showed a high affinity for the embedded L-Pen, which endowed the imprinted cavities with a larger number of sites and improved the selectivity toward Pen enantiomers. Under the optimal working conditions, the current response of the MIP/dsDNA sensor exhibited a positive linear relationship with the logarithm of the L-Pen concentration in the range of 3.0 × 10^−16^ to 3.0 × 10^−13^ mol/L, and the detection limit was 2.48 × 10^−16^ mol/L. After the introduction of dsDNA into the MIP, the selectivity of the sensor toward D-Pen increased by 6.4 times, and the sensor was successfully applied in the analysis of L-Pen in penicillamine tablets.

## 1. Introduction

Many endogenous macromolecular substances, such as enzymes, carriers, receptors, plasma proteins, and polysaccharides, have chiral characteristics [1]. The excessive biochemical reactions of commonly used drugs are related to the recognition and change of chirality. However, different enantiomers can have different pharmacological activities, toxicity, absorption, distribution, and metabolism [2]. Generally, only one enantiomer of a certain drug is effective against the target disease, while the other enantiomer either has toxic side effects or is ineffective and may cause harm to human health [3]. Therefore, the correct separation, detection, and analysis of chiral drugs are essential for protecting human health by reducing toxicity, side effects and dosage.

Molecularly imprinted polymers (MIPs) possess specific recognition and memory functions for specific target molecules [4,5]. Those materials exhibit not only high selectivity similar to that of biological antibody-antigen recognition, but also good chemical stability and mechanical strength, and they have been widely used in the field of chiral separation and analysis [6,7,8]. To further improve the chiral recognition ability of imprinted polymers, some studies have introduced macrocyclic compounds [9], polysaccharide [10], modified functional monomers [11], chiral metal complexes [12], and other materials for the preparation of polymers. However, the above methods possess certain disadvantages, such as limited scope of application and excessively strict molecular structure requirements; moreover, their chiral separation and recognition efficiency is not satisfactory.

To further improve these aspects, it is necessary to endow the imprinted membranes with the ability to induce and immobilize the molecular conformation. Studies have shown that drug molecules can bind to duplex DNA structures through intercalation binding, groove binding, and electrostatic binding [13,14,15]. Depending on the functional groups and conformational characteristics of the molecule, as well as the size or base gaps in the double-stranded deoxyribonucleic acid (dsDNA), an ordered spatial arrangement is occurred [16]. Otherwise, different forms of DNA have different stereo-conformational selectivity; for this reason, it is important to screen for DNA with a compatible chiral molecular conformation.

**Scheme 1:**
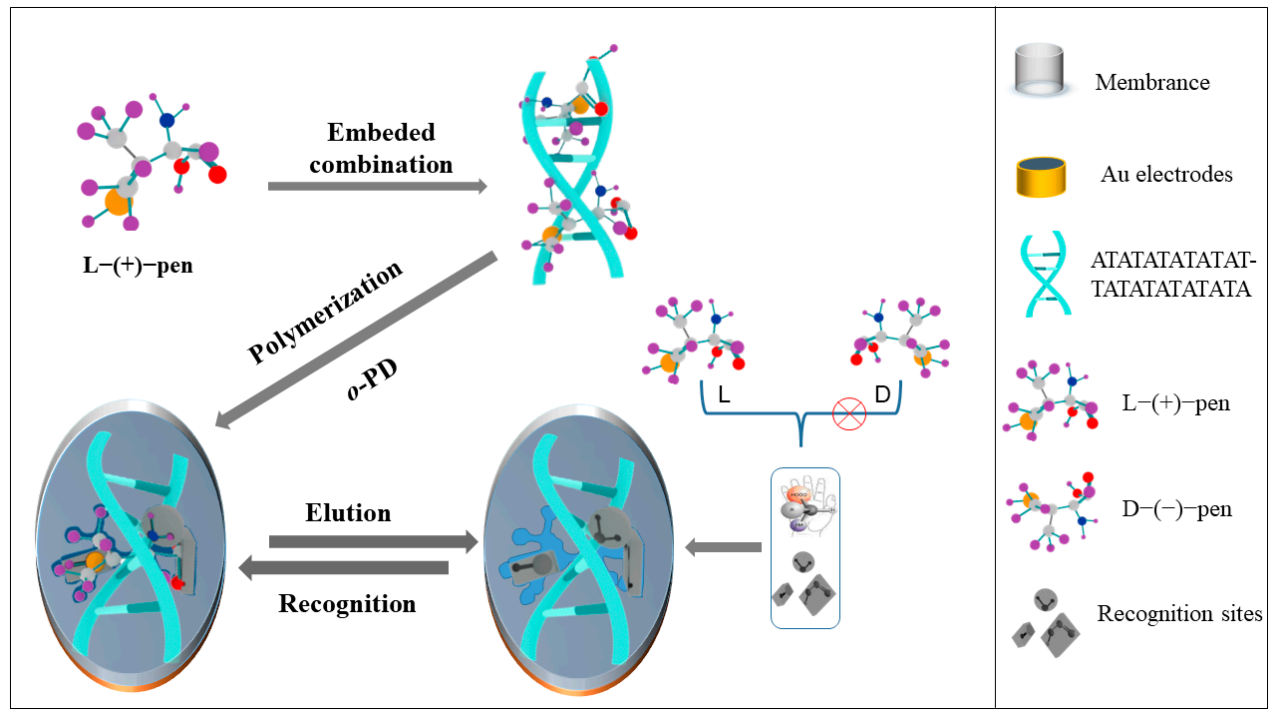
Principle of molecularly imprinted sensor for DNA immobilized L-pen conformation.

Penicillamine is a metabolite of penicillin with two enantiomers, D and L [17]. D-penicillamine (D-Pen) can treat Wilson’s disease and rheumatoid arthritis [18,19,20], and it has been used as an antidote for poisoning from copper and lead, among others [21,22,23]. In contrast, studies have found that L-penicillamine (L-Pen) is toxic and can cause neuritis and marrow damage [24,25]. Currently, the methods for detection and isolation of penicillamine involve flow injection [26], chromatography [27,28,29], spectrometry [30,31,32], colorimetric method [33], and electrochemical method [34,35,36,37]. However, the electrochemical sensor is miniaturized and easy to prepare, which effectively avoids the defects such as complex sample preparation, long detection time and expensive chiral selector. Therefore, it is significantly to establish a simple and highly selective electrochemical method for the detection of L-Pen. Here, we propose the use of optimized dsDNA as a chiral recognition element to induce chiral conformation and introduce it into MIPs: these are endowed with a triple-recognition ability through dsDNA-induced immobilized conformation, functional groups for base-assisted recognition, and recognition cavities formed by dsDNA-target molecule complexes. As shown in Scheme 1, the selected dsDNA was self-assembled on the surface of an Au electrode; then, L-Pen was inserted in the dsDNA for the subsequent electropolymerization to obtain L-Pen cavities of a highly specific conformation; finally, electrochemical methods were used to assess the sensor performance.

## 2. Materials and Methods

### 2.1. Apparatus and Reagents

A PGSTAT128N Autolab electrochemical workstation (Metrohm Co., Ltd., Herisau, Switzerland) with a three-electrode system was employed to study the electrochemical performance of the modified electrode. The MIP-modified Au electrode (Φ = 2 mm) was used as the working electrode, Ag/AgCl (saturated KCl) as the reference electrode, and Pt wire as the auxiliary electrode. A UV–vis spectrophotometer (UV-2400PC, Beijing Purkinje General Instrument Co., Ltd., Beijing, China), DYY-6C agarose gel electrophoresis (AGE) apparatus (Beijing Liuyi Biotechnology Co., Ltd., Beijing, China), and CHI660D electrochemical workstation (Shanghai Chenhua Instrument Co., Ltd., Shanghai, China) were also employed to study the performance of L-Pen intercalated into dsDNA. Liquid chromatography-mass spectrometry (LC-MS) of LCMS-9030 was used to evaluate the accuracy of the MIP/dsDNA sensor to detect L-Pen in real samples (Shimadzu, Co., Ltd., Suzhou, China).

L-Pen and D-Pen standards were purchased from Aladdin (Shanghai, China). The DNA sequence was customized by Shanghai Bioengineering Engineering Company, as shown in Table S1. Tris-EDTA buffer solution (TE) buffer, agarose, tris-(2-carboxyethyl) phosphine (TCEP, 10 mmol/L), tris Acetate-EDTA buffer solution, ethidium bromide, and 6.0 × glycerol gel were purchased from Shanghai Biotech Engineering Co., Ltd., while *o*-phenylenediamine (*o*-PD) was purchased from Aladdin (Shanghai, China). The probe molecule was 0.005 mol/L K_4_[Fe(CN)_6_]/K_3_[Fe(CN)_6_] containing 0.1 mol/L KCl solution. Methanol, ethanol, acetic acid, disodium hydrogen phosphate, sodium dihydrogen phosphate, and potassium chloride were purchased from Sichuan Xilong Science Company (Chengdu, China). Ultrapure water was used in all experiments (Sichuan Dubot Technology Co., Ltd., Sichuan, China).

### 2.2. Assay Procedures

The Au electrode was successively polished in chamois leather with alumina powder of particle diameter 1.0, 0.5, and 0.03 μm, and the impurities on the surface of the electrode were removed through ultrasonic cleaning with HNO_3_-H_2_O (*v*/*v*, 1/1), ethanol, and water for 5 min. TCEP (180 μL) was added to 20 μL of thawed ssDNA (5′-SH-C_6_-ATATATATATAT-3′) to activate the disulfide bond at room temperature (37 °C) for 2 h. The self-assembly process by Au-SH bond combination was carried out by immersing the Au electrode into a 1.0 × 10^−6^ mol/L solution of ssDNA. The modified electrode was then transferred into a dscDNA (3′-C_6_-TATATATATATA-5′) solution to perform the hybridization reaction (2 h; 37 °C) to form dsDNA. The dsDNA-modified electrode was transferred to a 1.0 × 10^−5^ mol/L solution of L-Pen for 21 min to obtain the dsDNA-L-Pen complex. Subsequently, the MIP/dsDNA-modified electrode was prepared by electropolymerization in a 5.0 × 10^−4^ mol/L *o*-PD solution (cyclic voltammetry (CV) parameters was performed for 15 cycles in a potential range from 0 V to 0.8 V at 50 mV/s). The MIP/dsDNA-modified electrode was placed in a methanol-acetic acid (*v*/*v*, 8/1; pH 8.0) solution under slight stirring for 20 min to elute the L-Pen template molecule, and the imprinted sensor for the induction and recognition of L-Pen conformation was obtained.

The preparation process of the non-MIP/dsDNA sensor was the same as that of the MIP/dsDNA sensor, but without the addition of L-Pen.

To compare the recognition capability of MIP/dsDNA, an MIP/non-dsDNA sensor was prepared. A bare Au electrode was placed in 10 mL of a mixture containing 5.0 × 10^−4^ mol/L *o*-PD and 1.0 × 10^−5^ mol/L L-Pen to prepare conventional MIPs by electropolymerization for 15 cycles. Then, the MIP/non-dsDNA-modified electrode was placed in a methanol-acetic acid (*v*/*v*, 8/1) solution for 20 min to elute the L-Pen target molecule; in this way, an MIP/non-dsDNA sensor with imprinted cavities matching L-Pen was obtained.

### 2.3. Measurement Methods

The electrochemical performance of the sensors was studied through differential pulse voltammetry (DPV) and CV in a 0.005 mol/L K_4_[Fe(CN)_6_]/K_3_[Fe(CN)_6_] solution (containing 0.1 mol/L KCl) with a potential ranging from +0.6 V to −0.2 V and a scanning speed of 50 mV/s. Electrochemical impedance spectroscopy (EIS) was performed at the frequency range from 0.1 Hz to 100 kHz, with an alternating voltage of 5 mV. TE buffer (pH 8.0) was employed for background subtraction. UV–vis spectroscopy, with a scanning range from 220 nm to 320 nm and a scanning interval of 0.1 nm, was used to study the interaction mode of dsDNA and L-Pen. AGE (working voltage: 60 V) was carried out to investigate the changes before and after the reaction of each dsDNA type with L-Pen.

## 3. Results and Discussion

### 3.1. Interaction between L-Pen and dsDNA

UV–vis spectroscopy was used to study the interaction between dsDNA and the L-Pen molecule. A 1.0 × 10^−5^ mol/L L-Pen solution was added to five types of dsDNA and stirred overnight to react completely. The change in the maximum absorption wavelength (λmax) of dsDNA after interaction with L-Pen was recorded. The results are presented in Figure S1 (Supplementary Materials), which shows that λmax red-shifted by approximately 5 nm following the interaction between dsDNA and L-Pen, indicating that the interaction mechanism between the two species was intercalation binding [13].

The electrochemical method was used to study the intercalation time of five types of dsDNA with L-Pen. As shown in Figure S2, three structures (dsDNA_1_, AAAAAAAAAAAA-TTTTTTTTTTTT, 7 min; dsDNA_2_, ACACACACACACACAC-TGTGTGTGTGTG, 2.5 min; and dsDNA_3_, AGAGAGAGAGAG-TCTCTCTCTCTC, 7 min) were saturated with L-Pen in a short time, which suggests that they all possessed large cavities capable of exerting a weak steric hindrance toward L-Pen, thereby facilitating the intercalation. This implies that the interference of analogues, in these cases, would not be eliminated effectively. The interaction of L-Pen with dsDNA_5_ (CGCGCGCGCGCG-GCGCGCGCGCGC, 35 min) reached saturation over a long time: this suggests that the structure cavity of dsDNA_5_ presented a strong steric hindrance, obstructing L-Pen entry and resulting in a low recognition and separation efficiency. In contrast, in dsDNA_4_ (ATATATATATAT-TATATATATATA), the response current of the probe decreased slowly with increasing time, but stabilized at 19 min, indicating a saturated interaction between dsDNA_4_ and L-Pen. Therefore, dsDNA4 was selected as the element to immobilize the L-Pen conformation and ensure ultra-high selectivity and capture performance of the MIP/dsDNA sensor.

AGE was used to study changes in mobility and combine performance of each dsDNA and L-Pen. The results are shown in Figure S3, where k is a DNA marker with clear levels to indicate the molecular weight of DNA from top to bottom on the agarose gel; letters from (a) to (e) indicate the dsDNA structures from 1 to 5 in sequence, which had approximately the same molecular weight; and letters (f) to (j) indicate the smearing after the interaction of each dsDNA with L-Pen. After the interaction between L-Pen and dsDNA_1_, dsDNA_2_, and dsDNA_3_, the molecular sizes of the three DNA structures increased and shifted slightly in AGE, indicating a weak steric hindrance toward L-Pen; therefore, the interference from analogues could be effectively eliminated. Furthermore, after the separate reactions of dsDNA_4_ and dsDNA_5_ with L-Pen, dsDNA_5_ moved over a longer distance due to a smaller friction force compared to that experienced by dsDNA_4_ in AGE: this suggests that dsDNA_5_ was characterized by a more significant steric hindrance and difficult interaction with L-Pen. However, the combination of dsDNA_4_ and L-Pen was relatively stable, and the internal structure matched the conformation of L-Pen, which assisted in its recognition. Therefore, the AGE experiment confirmed dsDNA_4_ as a useful element for the auxiliary recognition of L-Pen.

### 3.2. Electrochemical Characterization of MIP/dsDNA and Non-MIP/dsDNA

**Figure 1:**
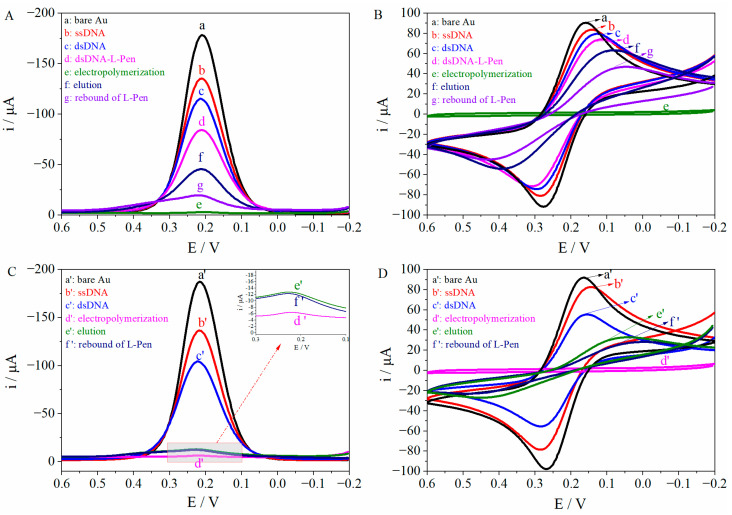
The DPV (**A**) and CV (**B**) responses of MIPs/dsDNA under different conditions and the DPV (**C**) and CV (**D**) of non-MIPs/dsDNA under different conditions (working potential: −0.2~0.6 V; scan rate: 50 mV/s; pulse amplitude: 50 mV).

The properties of MIP/dsDNA in different processes were studied using DPV and CV. As shown in Figure 1A, curve a represents the bare Au electrode; when the electrode was modified by the self-assembly of ssDNA (curve b), hybridization (curve c), and embedding (curve d), the current intensity gradually decreased because the modification element impeded electron transport. After electropolymerization, a dense and weakly conductive MIP/dsDNA film was formed on the surface of the electrode, which significantly hindered the transfer of electrons and caused the current intensity of the probe to decrease sharply (curve e). A mixed solution of methanol-acetic acid (*v*/*v*, 8/1) was used to elute L-Pen from the polymer for 20 min, forming the corresponding conformational cavities, and the current intensity of the probe rebounded (curve f). Then, when the sensor was placed in a 5.0 × 10^−14^ mol/L L-Pen solution for 16 min, the size, structure, and recognition sites of the L-Pen-imprinted MIP/dsDNA cavities were stably filled again. This resulted in the blockage of electron transfer channels, and the peak current decreased (curve g). Therefore, quantitative analysis of L-Pen was successfully performed. The results were consistent with those obtained by CV (Figure 1B), further supporting the successful fabrication of the MIP/dsDNA sensor capable of L-Pen recognition.

The performance of non-MIP/dsDNA under different process conditions was also studied using DPV, as shown in Figure 1C, where curve a’ represents the bare Au electrode. The current intensity of the probe gradually decreased from curve a’ to d’: this was attributed to the action of self-assembled dsDNA and non-MIP/dsDNA film, which gradually hindered electron transport on the electrode surface. As L-Pen was not present in the non-MIP/dsDNA, there were no imprinted cavities to recognize L-Pen after a 20 min elution (curve e’) with a mixed solution of methanol-acetic acid (*v*/*v*, 8/1; pH 8.0). Thus, the rebound signal of L-Pen (5.0 × 10^−14^ mol/L, 16 min) remained substantially unchanged (curve f’). These results, in agreement with the CV responses (Figure 1D), proved that the non-MIP/dsDNA sensor could not recognize L-Pen, eliminating the possibility of the physical adsorption of L-Pen to MIP/dsDNA

**Figure 2:**
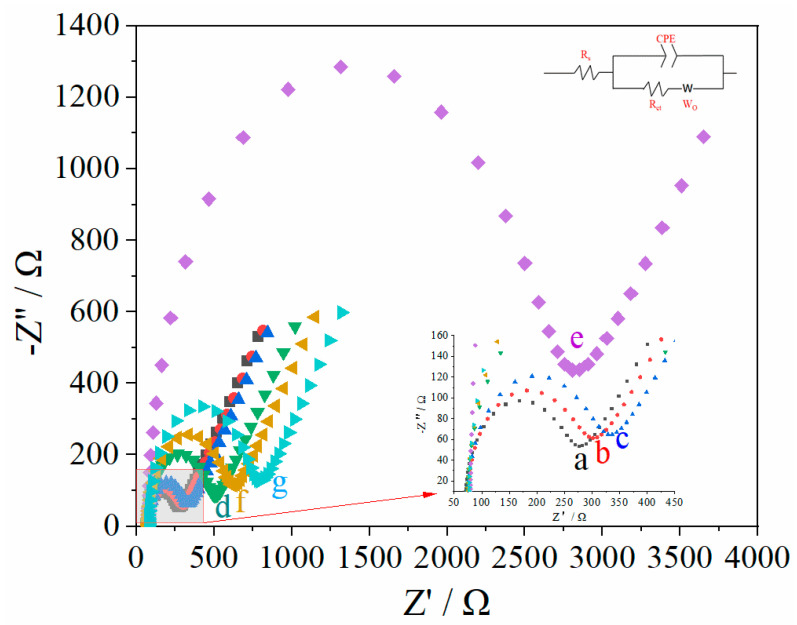
The EIS responses of MIPs/dsDNA sensor under different conditions in 0.005 mol/L of K_4_[Fe(CN)_6_]/K_3_[Fe(CN)_6_] (contain 0.1 mol/L KCl) solution over the frequency range from 0.1 Hz to 100 kHz, with an alternating voltage of 5 mV (a: bare Au electrode, b: ssDNA, c: dsDNA, d: dsDNA-L-Pen, e: electropolymerization, f: elution, g: rebound).

EIS was used to evaluate the electrode modification process in a 0.005 mol/L K_4_[Fe(CN)_6_]/K_3_[Fe(CN)_6_] solution as probe, as shown in Figure 2. The processes of self-assembly (b, 201.1 Ω) and hybridization (c, 229.2 Ω) were sequentially performed on the surface of bare Au electrode (a, 183.4 Ω), which promoted a gradual increase in resistance. When L-Pen was embedded with dsDNA structure, the corresponding resistance value increased again (d, 390.3 Ω). After electropolymerization, a dense and weakly conductive MIPs/dsDNA film severely impeded the probe transfer on the electrode surface, and the resistance value increased sharply (e, 2508.0 Ω). A methanol-acetic acid (*v*/*v*, 8/1) mixture was used to elute some L-Pen from the polymer for 20 min; the obtained imprinted cavities acted as transport channels of the probe, and the resistance value decreased (f, 492.2 Ω). When the MIP/dsDNA sensor rebounded with 5.0 × 10^−14^ mol/L mol/L L-Pen for 16 min, the L-Pen-imprinted cavities were stably filled again, thereby increasing the resistance again (g, 649.7 Ω). EIS curve fitting, carried out with ZsimpWin software, showed that the fitted curves of the equivalent circuit diagram R(C(RW)) were consistent with the measured curves under the same conditions.

The EIS of 5.0 × 10^−14^ mol/L L-Pen re-adsorbed by this sensor is fitted by software ZsimpWin, the solution resistance *R_s_* is 77.03 Ω, the double layer capacitance (CPE) is 2.559 × 10^−6^, and the actual resistance (*R_ct_*)of molecularly imprinted film is 649.7 Ω. According to the Nyquist equation and the following formula,

(1) $${\left(Z^{\prime }-R_{s}-\frac{R_{ct}}{2}\right)}^{2}+{Z^{\prime \prime }}^{2}={\left(\frac{R_{ct}}{2}\right)}^{2}$$

(2) $$\tan\Phi =\frac{-Z^{\prime \prime }}{Z^{\prime }}$$

(3) ω=2πf 

(4) $$Z^{\prime }=R_{s}+\frac{R_{ct}}{1+\omega^{2}C_{d}{}^{2}R_{ct}{}^{2}}$$

(5) $$Z^{\prime \prime }=-\frac{\omega C_{d}R_{ct}{}^{2}}{1+\omega^{2}C_{d}{}^{2}R_{ct}{}^{2}}\;$$

(6) $$Z=Z^{\prime }-jZ^{\prime \prime }$$

When the frequency is 86.8496 Hz, the real part of impedance *Z*′ is 376.7 Ω, the imaginary part *Z*′ is 296.9 Ω, the solution resistance *R_s_* is 71.1 Ω, the phase angle *Φ* is 38.2°, the double-layer capacitance CPE is 2.4324 × 10^−6^, and the actual impedance *R_ct_* of molecularly imprinted film is 594.1 Ω. This is basically consistent with the results of the software simulation and impedance diagram.

### 3.3. Optimization of the MIP/dsDNA Sensor

**Figure 3:**
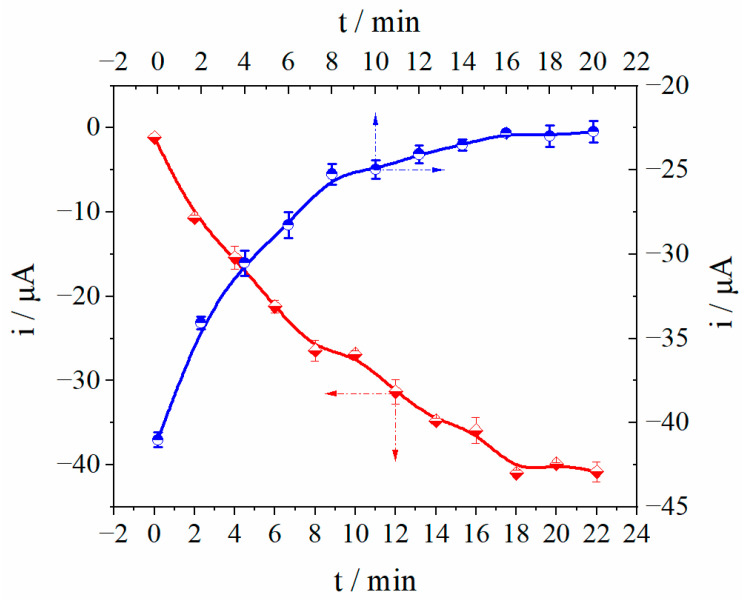
Optimization of elution time (**a**) and rebound time (**b**).

The detection of L-Pen by MIP/dsDNA sensor was mainly affected by the following different factors, including the binding time of dsDNA and L-Pen, the number of electropolymerization cycles, eluent, pH value, elution time and rebound time. Therefore, the above parameters were optimized, respectively. The results show that with the combination time of dsDNA and L-Pen of 21 min (Figure S2), 15 cycles of electropolymerization (Figure S4), methanol-acetic acid (*v*/*v*, 8/1; pH 8.0) as eluent (Figure S5), elution time of 20 min (Figure 3a), and rebound time of 16 min (Figure 3b), the MIP/dsDNA sensor has the best detection performance for L-Pen.

### 3.4. DPV Response of the MIP/dsDNA Sensor to Identify L-Pen at Different Concentrations

**Figure 4:**
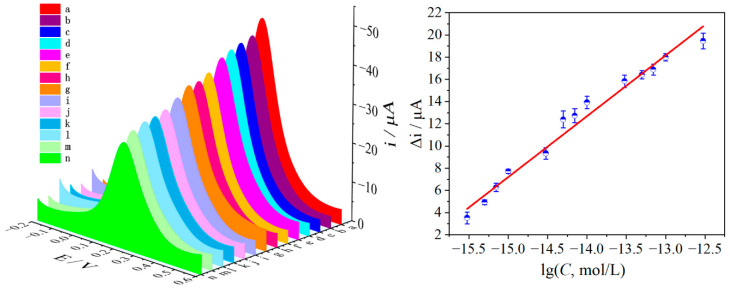
The DPV response of MIPs/dsDNA sensor to L-Pen on the 0.005 mol/L K_4_[Fe(CN)_6_]/K_3_[Fe(CN)_6_] (contain 0.1 mol/L KCl) and corresponding calibration curves (a~n: the concentration of L-Pen were 0, 3.0 × 10^−16^, 5.0 × 10^−16^, 7.0 × 10^−16^, 1.0 × 10^−15^, 3.0 × 10^−15^, 5.0 × 10^−15^, 7.0 × 10^−15^, 1.0 × 10^−14^, 3.0 × 10^−14^, 5.0 × 10^−14^, 7.0 × 10^−14^, 1.0 × 10^−13^, 3.0 × 10^−13^ mol/L L-Pen) (working potential from +0.6 V to −0.2 V. Error bar represents the standard deviation of three repetitive experiments).

The recognition ability of the MIP/dsDNA sensor toward L-Pen was studied under the optimized conditions. The sensor was immersed in L-Pen solutions at different concentrations, and DPV signals were recorded to evaluate the relationship between the concentration of L-Pen and current intensity. As shown in Figure 4, the DPV response signals gradually decreased as the L-Pen concentration increased, indicating that more L-Pen rebounded to the imprinted cavity. The current signal responses of the MIP/dsDNA sensor exhibited a positive linear relationship with the L-Pen concentration logarithm in the range from 3.0 × 10^−16^ to 3.0 × 10^−13^ mol/L. Such a relationship is described by the linear equation Δi (μA) = 5.48 × 10^−6^ lg (C, mol/L) + 8.94 × 10^−5^ (r = 0.993). The detection limit (DL) was 2.48 × 10^−16^ mol/L (DL = 3δ_b_/K, where DL is the detection limit at 95% confidence level; δ_b_ is the standard for blank sample detection Quasi-deviation; K is the slope of the working curve). Compared with the performance of other methods (Table S2), the MIP/dsDNA sensor was more sensitive in the separation and recognition of L-Pen and showed a lower DL. This is because the imprinted cavity structure was improved by dsDNA, which provided a larger number of recognition sites to meet the accuracy and sensitivity requirements of chiral recognition and separation.

### 3.5. Chiral Separation and Recognition Performance of the Sensors

**Figure 5:**
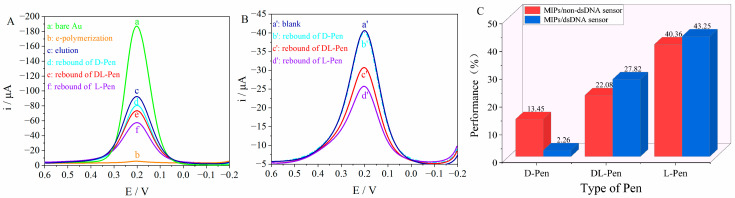
The DPV responses of MIPs/non-dsDNA sensor (**A**) and MIPs/dsDNA sensor (**B**) on 5.0 × 10^−14^ mol/L of Penicillamine enantiomers in 0.005 mol/L of K_4_[Fe(CN)_6_]/K_3_[Fe(CN)_6_] (contain 0.1 mol/L KCl) solution over a potential range from +0.6 V to −0.2 V, with a scan rate of 50 mV/s and pulse amplitude of 50 mV; the recognition performance (**C**) of MIPs/dsDNA sensor and MIPs/non-dsDNA sensor for Pen enantiomers.

To evaluate the performance of the MIP/dsDNA sensor in the chiral recognition and separation of the Pen enantiomers, a MIP/non-dsDNA sensor was prepared for comparison. A 5.0 × 10^−14^ mol/L solution of Pen enantiomers was selected as the detection target, and the DPV signal of the sensor was recorded. The results are shown in Figure 5A, where the eluted signal (curve c) was used as a blank, and the MIP/non-dsDNA sensor rebounds were those for D-Pen (curve d), DL-Pen (curve e), and L-Pen (curve f). The probe response signal decreased gradually: the response to D-Pen decreased by approximately 13.45%, while that to DL-Pen decreased by approximately 22.08%, highlighting the sensor’s poorly selective recognition ability to distinguish and separate Pen enantiomers. In the MIP/dsDNA tests (Figure 5B), where the eluted signal (curve a’) was used as a blank, the sensor only had a distinct response to DL-Pen (curve b’) and L-Pen (curve c’), while the signal corresponding to D-Pen (curve d’) decreased by 2.26%, and that of DL-Pen decreased by 27.82%. The selectivity of this sensor to D-Pen was 6.4 times higher than that of the MIP/non-dsDNA one. This indicates that the fine structure of the imprinted recognition sites can be improved by introducing embedded dsDNA to immobilize the spatial conformation of the chiral molecule. This supports the possibility of improving the chiral recognition ability of the molecularly imprinted technology. The recognition performance of the MIP/dsDNA and MIP/non-dsDNA sensors for the Pen enantiomers is shown in Figure 5C.

**Figure 6:**
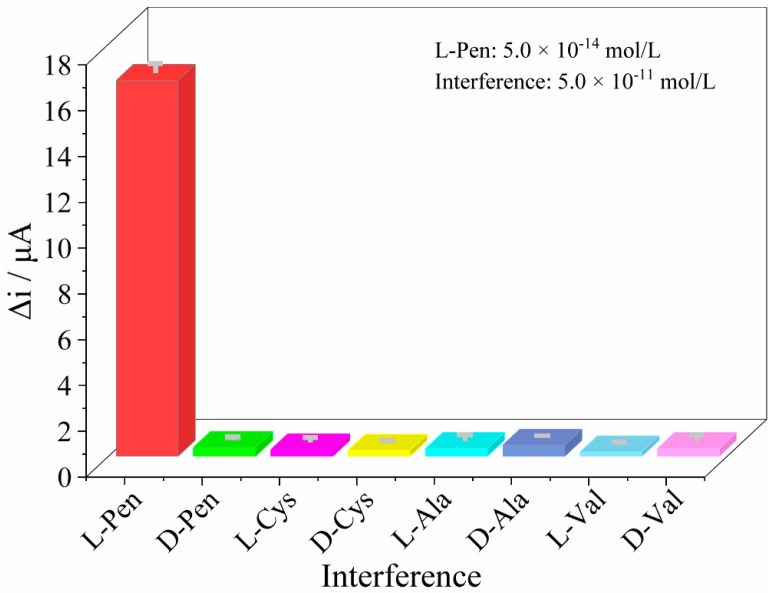
The current response of the sensor to the similar chiral substance of L-Pen (the concentration of L-Pen was 5.0 × 10^−14^ mol/L and the interference concentration was control as 5.0 × 10^−11^ mol/L).

The selectivity of the MIP/dsDNA sensor was assessed by rebounding interfering substances such as L-Valine (L-Val), D-Valine (D-Val), L-Cysteine (L-Cys), D-Cysteine (D-Cys), L-Alanine (L-Ala), D-Alanine (D-Ala), and D-Pen at a concentration of 5.0 × 10^−11^ mol/L and by recording the DPV signals to compare them with the rebounding of 5.0 × 10^−14^ mol/L L-Pen. The results are shown in Figure 6, where the highest interfering signal (D-Ala) only accounts for 2.98% of the L-Pen response signal, suggesting the high selectivity of the sensor in the presence of analogue substances.

### 3.6. Stability and Reproducibility

The reproducibility of the sensor is one of the most important issues in electrochemical sensor fabrication. Herein, the reproducibility was evaluated by recording the DPV signal of the prepared sensor after rebinding with 5.0 × 10^−14^ mol/L L-Pen. The renewal of the sensor was achieved by removing the templates in a methanol-acetic acid (*v*/*v*, 8/1; pH = 8.0) mixture. The relative standard deviation (RSD) of the eight measurements was 1.40 %. In addition, five sensors were prepared and used to measure 5.0 × 10^−14^ mol/L L-Pen under the same conditions; an RSD of 2.64% was obtained, reflecting an acceptable sensor-to-sensor reproducibility. The stability of the sensor was also examined. When the prepared sensors were not in use, they were stored at 4 °C; we observed that 95.5% of the initial response was retained after 14 days, indicating acceptable sensor stability.

### 3.7. Sample Detection

The applicability and reliability of the MIP/dsDNA sensor were evaluated through the assay of L-Pen in drugstore penicillamine tablets (The weight of penicillamine tablets is 345 mg, in which the labeled amount is 125 mg penicillamine, which contains about 99.95% or more D-enantiomer). After removing and finely grinding the penicillamine tablets, 0.375 g of powder was weighed and dissolved in a 250 mL volumetric bottle. After filtration, 10 mL of the filtrate was brought to 100 mL using ultrapure water for testing. The sensor was immersed in the sample solution for the rebounding of L-Pen, and the DPV current was recorded. In addition, in order to further compare the determination results of MIP/dsDNA sensor, LC-MS was used for comparison. Because the detection limit of LC-MS does not meet the requirements of the determination of ultra-trace L-Pen, the pretreated samples are concentrated 1000 times before determination. Table 1 shows that the recovery rates of the sensor range from 96.20% to 103.43%, which proves the applicability of the sensor.

## 4. Conclusions

In this paper, a novel molecularly imprinted chiral sensing platform for the highly selective ultra-trace detection of L-Pen was developed. Based on dsDNA as the functional unit of directional recognition of L-Pen enantiomers in MIP films, the dual recognition of L-Pen by dsDNA and MIP membranes was realized. Due to the anchoring of dsDNA in the MIP membrane, the stereoselectivity of MIP membrane to L-Pen was significantly improved, and the interference of D-Pen and chiral analogues was eliminated. Furthermore, the structural diversity of dsDNA and its specific binding modes with molecules, such as intercalation binding, groove binding, electrostatic binding, etc., greatly expand the applicability of MIP/dsDNA sensor without being limited to the structure and functional groups of chiral molecules. This method has excellent application prospects and can meet the requirements of ultra-trace chiral separation and detection in the fields of biomedicine, chiral pollution, pesticide residue analysis, medical diagnosis and so on.

## Figures and Tables

**Table 1:** The results of MIPs/dsDNA sensor detection of L-Pen in penicillamine tablets.

Samples	Found 10^−14^ mol/L	RSD% n = 7	Added 10^−14^ mol/L	Total Found 10^−14^ mol/L	RSD% n = 7	Recoveries%	LC-MS 10^−14^ mol/L
I	5.82	4.42	3.00	8.79	4.04	98.91	5.79
II	6.24	3.56	6.00	12.00	3.16	96.20	6.01
III	5.45	4.32	6.00	11.65	3.98	103.43	5.52

## Data Availability

The data presented in this study are available on request from the corresponding author.

## Supplementary Materials

The following supporting information can be downloaded at: https://www.mdpi.com/article/10.3390/polym14194133/s1, Figure S1: UVs of 1.0 × 10^−5^ mol/L of L-Pen embedded in different dsDNA, TE buffer as solvent (pH 8.0). The dsDNA sequences were dsDNA_1_ (AAAAAAAAAAAA-TTTTTTTTTTTT), dsDNA_2_ (ACACACACACAC-TGTGTGTGTGTG), dsDNA_3_ (AGAGAGAGAGAG-TCTCTCTCTCTC), dsDNA_4_ (ATATATATATAT-TATATATATATA) and dsDNA_5_ (CGCGCGCGCGCG-GCGCGCGCGCGC) respectively; Figure S2: The effect embedding time of 1.0 × 10^−5^ mol/L of L-Pen embedded in different dsDNA in 0.005 mol/L of K_4_[Fe(CN)_6_]/K_3_[Fe(CN)_6_] (contain 0.1 mol/L KCl) solution over a potential range from +0.6 V to −0.2 V, with a scan rate of 50 mV/s and pulse amplitude of 50 mV; Figure S3: Characterization of L-Pen treated by each double-stranded DNA by AGE (a: dsDNA_1_, b: dsDNA_2_, c: dsDNA_3_, d: dsDNA_4_, e: dsDNA_5_, f: dsDNA_1_-L-Pen, g: dsDNA_2_-L-Pen, h: dsDNA_3_-L-Pen, i: dsDNA_4_-L-Pen, j: dsDNA_5_-L-Pen, k: DNA marker); Figure S4: The formation of MIPs/dsDNA via electropolymerization (Scan rate:50 mV/s; cycle number: 15; working potential: 0~0.8 V); Figure S5: The Effect of eluent pH value on MIP/dsDNA sensor recognition of L-Pen; Table S1: DNA base sequences; Table S2: Comparison of performance between this method and other methods for detecting L-Pen. References [24,30,31,32,33,34,35,36,37] are cited in the Supplementary Materials.

## References

[1] Malyshko E.V., Tverdislov V.A. (2016). Chirality as a physical aspect of structure formation in biological macromolecular systems. J. Phys. Conf. Ser.

[2] Zhou Q., Yu L.S., Zeng S. (2014). Stereoselectivity of chiral drug transport: A focus on enantiomer–transporter interaction. Drug Metab. Rev..

[3] Singh N., Sharma L. (2018). Enantioseparation of D-and L-isomers of chiral drugs for improving their bioavailability: Some techniques including micellization with gemini surfactants. Indian J. Pharm. Educ..

[4] Uzun L., Turner A.P.F. (2016). Molecularly-imprinted polymer sensors: Realising their potential. Biosens. Bioelectron..

[5] Tiwari M.P., Prasad A. (2015). Molecularly imprinted polymer based enantioselective sensing devices: A review. Anal. Chim. Acta.

[6] Rutkowska M., Płotka-Wasylka J., Morrison C., Wieczorek P.P., Namieśnik J., Marć M. (2018). Application of molecularly imprinted polymers in analytical chiral separations and analysis. Trends Anal. Chem..

[7] Li D., Luo K., Zhang L.M., Gao J.X., Liang J.L., Li J.P., Pan H.C. (2021). Research and Application of Highly Selective Molecular Imprinting Technology in Chiral Separation Analysis. Crit. Rev. Anal. Chem..

[8] Yang S., Wang Y., Jiang Y.D., Li S., Liu W. (2016). Molecularly imprinted polymers for the identification and separation of chiral drugs and biomolecules. Polymers.

[9] Wu T., Wei X.P., Ma X.H., Li J.P. (2017). Amperometric sensing of L-phenylalanine using a gold electrode modified with a metal organic framework, a molecularly imprinted polymer, and β-cyclodextrin-functionalized gold nanoparticles. Microchim. Acta.

[10] Lin L., Lian H.T., Sun X.Y., Yu Y.M., Liu B. (2015). An L-dopa electrochemical sensor based on a graphene doped molecularly imprinted chitosan film. Anal. Methods.

[11] Alizadeh T., Bagherzadeh A., Shamkhali A.N. (2016). Synthesis of nano-sized stereoselective imprinted polymer by copolymerization of (S)-2-(acrylamido) propanoic acid and ethylene glycol dimethacrylate in the presence of racemic propranolol and copper ion. Mater. Sci. Eng. C.

[12] Chen X.H., Zhang S.B., Shan X.L., Chen Z.D. (2019). Derivative chiral copper (II) complexes as template of an electrochemical molecular imprinting sol-gel sensor for enantiorecognition of aspartic acid. Anal. Chim. Acta.

[13] Rehman S.U., Sarwar T., Husain M.A., Ishqi H.M., Tabish M. (2015). Studying non-covalent drug–DNA interactions. Arch. Biochem. Biophys..

[14] Feagin T.A., Olsen D.P.V., Headman Z.C., Heemstra J.M. (2015). High-throughput enantiopurity analysis using enantiomeric DNA-based sensors. J. Am. Chem. Soc..

[15] Doan P., Pitter D.R.G., Kocher A., Wilson J.N., Goodson T. (2016). A new design strategy and diagnostic to tailor the DNA-binding mechanism of small organic molecules and drugs. ACS Chem. Biol..

[16] Zhang L.M., Zhang D.Y., Zeng Y., Li J.P. (2018). A Cimaterol Molecularly Imprinted Sensor Based on DNA-assisted Recognition. Chin. J. Anal. Chem..

[17] Zawawi R.M., Zheng A.L.T. (2020). Zinc oxide/vancomycin-based electrochemical chiral sensor for the recognition of penicillamine enantiomers. Int. J. Electrochem. Sci..

[18] Devassy D.E., Harshad S.R., Devarbhai H. (2019). Pompholyx-like eruptions induced by penicillamine in a patient with wilson’s disease. Indian J. Dermatol..

[19] Valenzuela-Ubiña S., Jiménez-Gallo D., Russo-de la Torre F., Linares-Barrios M. (2020). Elastosis perforans serpiginosa induced by d-penicillamine treated with cyclosporine and allopurinol. Dermatol. Ther..

[20] Durán G.M., Abellán C., Contento A.M., Ríos Á. (2017). Discrimination of penicillamine enantiomers using β-cyclodextrin modified CdSe/ZnS quantum dots. Microchim. Acta.

[21] Jalali S.M., Najafzadeh H., Bahmei S. (2017). Protective role of silymarin and D-penicillamine against lead-induced liver toxicity and oxidative stress. Toxicol. Ind. Health.

[22] Aaseth J., Skaug M.A., Cao Y., Andersen O. (2015). Chelation in metal intoxication—Principles and paradigms. J. Trace Elem. Med. Biol..

[23] Sisombath N.S., Jalilehvand F., Schell A.C., Wu Q. (2014). Lead (II) binding to the chelating agent D-penicillamine in aqueous solution. Inorg. Chem..

[24] Wang Y.H., Han Q., Zhang Q., Huang Y.H., Guo L.J., Fu Y.Z. (2013). Chiral recognition of penicillamine enantiomers based on a vancomycin membrane electrode. Anal. Methods.

[25] Wilson J.E., Du Vigneaud V. (1950). Inhibition of the growth of the rat by L-penicillamine and its prevention by aminoethanol and related compounds. J. Biol. Chem..

[26] Kukoc-Modun L., Biocic M., Radie N. (2020). Flow-injection Determination of Glutathione, Penicillamine and Tiopronin Based on the Reduction of Copper (II)-neocuproine Reagent. Croat. Chem. Acta.

[27] Song L.J., Guo Z.P., Chen Y. (2012). Separation and determination of chiral composition in penicillamine tablets by capillary electrophoresis in a broad p H range. Electrophoresis.

[28] Sotgia S., Zinellu A., Forteschi M., Paliogiannis P., Deiana G.A., Pinna G.A., Mangoni A.A., Carru C. (2018). A liquid chromatography-mass spectrometry study on the spirocyclization of ninhydrin with the aminothiols. Microchem. J..

[29] Sotgia S., Zinellu A., Pinna G.A., Deiana L., Carru C. (2011). Application of an unusual ninhydrin-based reaction for the indirect chiral resolution of D, L-penicillamine. Talanta.

[30] Huang Y.M., Yang J.D., Yuan H.Y., Guo Y., Zeng X.Q., Cheng J.W., Zhang Y.H. (2018). A novel competitive-displacement fluorescence assay for l-penicillamine based on the reaction between the target and N-acetyl-l-cysteine-capped CdTe quantum dots for copper ions. Anal. Methods.

[31] Ngamdee K., Puangmali T., Tuntulani T., Ngeontae W. (2015). Circular dichroism sensor based on cadmium sulfide quantum dots for chiral identification and detection of penicillamine. Anal. Chim. Acta..

[32] Zhang Y., Wang H.Y., He X.W., Li W.Y., Zhang Y.K. (2021). Homochiral fluorescence responsive molecularly imprinted polymer: Highly chiral enantiomer resolution and quantitative detection of L-penicillamine. J. Hazard. Mater..

[33] Lian J.J., Liu P., Liu Q.Y. (2022). Nano-scale minerals in-situ supporting CeO_2_ nanoparticles for off-on colorimetric detection of L–penicillamine and Cu2+ ion. J. Hazard. Mater..

[34] Lin X., Zhu S., Wang Q.H., Xia Q., Ran P.Y., Fu Y.Z. (2016). Chiral recognition of penicillamine enantiomers using hemoglobin and gold nanoparticles functionalized graphite-like carbon nitride nanosheets via electrochemiluminescence. Colloids Surf. B.

[35] Wang Y.H., Han Q., Zhang Q., Huang Y.H., Guo L.J., Fu Y.Z. (2013). Enantioselective recognition of penicillamine enantiomers on bovine serum albumin-modified glassy carbon electrode. J. Solid State Electrochem..

[36] Wang Y.H., Zhou J., Han Q., Chen Q., Guo L.J., Fu Y.Z. (2012). Chiral Recognition of Penicillamine Enantiomers Based on DNA-MWNT Complex Modified Electrode. Electroanalysis.

[37] Wu X.F., Ge Q., Jiang N., Liu M., Cong H., Tao Z. (2022). Ultrasensitive sensor for L-penicillamine with chirality-induced amplification of benzo [3] uril electrochemiluminescence via supramolecular interactions. Sens. Actuators B.

