# Supplementary material for: DNA-Immobilized Special Conformation Recognition of L-Penicillamine Using a Chiral Molecular Imprinting Technique
Source: Polymers (Basel). 2022 Oct 2;14(19):4133. doi: 10.3390/polym14194133 (PMC9571851; doi:10.3390/polym14194133)
Supplement: Supplementary file 1 [file polymers-14-04133-s001.zip › polymers-1860620-supplementary.pdf]

Supplementary Materials

DNA-Immobilized Special Conformation Recognition of L-Penicillamine Using a Chiral Molecular Imprinting Technique

Lianming Zhang *, Kui Luo, Jingxia Gao and Jianping Li

College of Chemistry and Bioengineering, Guilin University of Technology, Guilin 541004, China; kuiluo95@126.com (K.L.); gaojingxia1111@163.com (J.G.); likianping@263.net (J.L.)

* Correspondence: lianming226@126.com; Tel.: +86-773-589-5622

1. Interaction between L-Pen and dsDNA

UV–vis spectroscopy was used to study the interaction between dsDNA and the L-Pen molecule. A 1.0 × 10^{-5} mol/L L-Pen solution was added to five types of dsDNA and stirred overnight to react completely. The change in the maximum absorption wavelength (λ_{max}) of dsDNA after interaction with L-Pen was recorded. The results are presented in **Figure S1**, which shows that λ_{max} red-shifted by approximately 5 nm following the interaction between dsDNA and L-Pen, indicating that the interaction mechanism between the two species was intercalation binding.

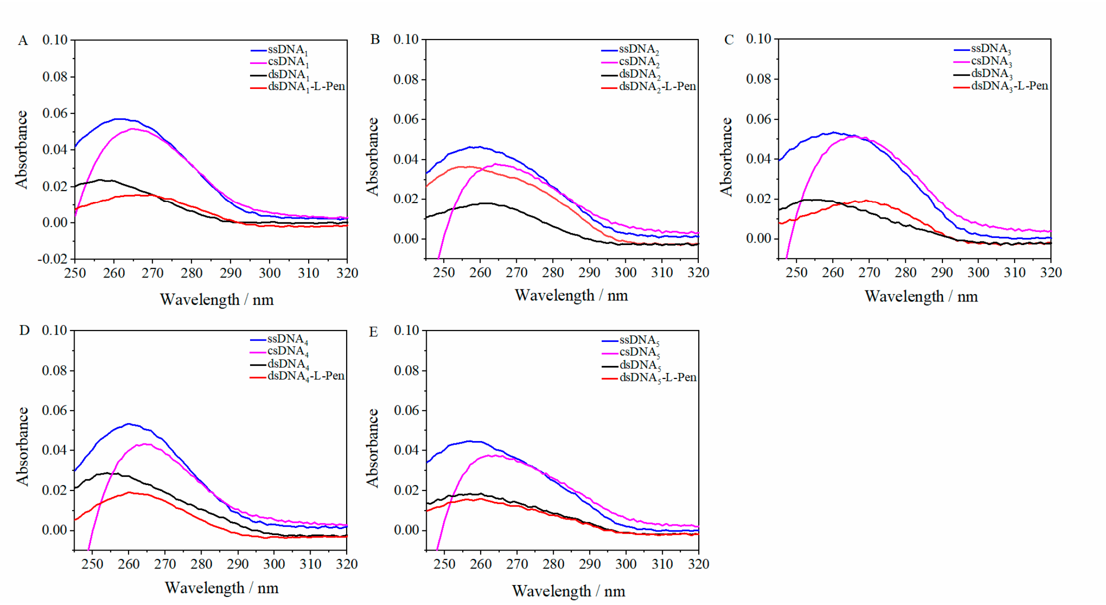

Figure S1. UVs of 1.0 × 10^{-5} mol/L of L-Pen embedded in different dsDNA, TE buffer as solvent (pH 8.0). The dsDNA sequences were $dsDNA_1$ (AAAAAAAAAAAA-TTTTTTTTTTTT), $dsDNA_2$ (ACACACACACAC-TGTGTGTGTGTG), $dsDNA_3$ (AGAGAGAGAGAG-TCTCTCTCTCTC), $dsDNA_4$ (ATATATATATAT-TATATATATATA) and $dsDNA_5$ (CGCGCGCGCGCG-GCGCGCGCGCGC) respectively.

The electrochemical method was used to study the intercalation time of five types of dsDNA with L-Pen. As shown in **Figure S2**, three structures ($dsDNA_1$, AAAAAAAAAAAA-TTTTTTTTTTTT, 7 min; $dsDNA_2$, ACACACACACACACAC-TGTGTGTGTGTG, 2.5 min; and $dsDNA_3$, AGAGAGAGAGAG-TCTCTCTCTCTC, 7 min) were saturated with L-Pen in a short time, which suggests that they all possessed large cavities capable of exerting a weak steric hindrance toward L-Pen, thereby facilitating the intercalation. This implies that the interference of analogues, in these cases, would not be eliminated effectively. The interaction of L-Pen with $dsDNA_5$ (CGCGCGCGCGCG-GCGCGCGCGCGC, 35 min) reached saturation over a long time: this suggests that the structure cavity of $dsDNA_5$ presented a strong steric hindrance obstructing L-Pen entry and resulting in a low recognition and separation efficiency. In contrast, in $dsDNA_4$ (ATATATATATAT-TATATATATATA), the response current of the probe decreased slowly with increasing time, but stabilized at 19 min, indicating a saturated interaction between

$dsDNA_4$ and L-Pen. Therefore, $dsDNA_4$ was selected as the element to immobilize the L-Pen conformation and ensure ultra-high selectivity and capture performance of the MIP/dsDNA sensor.

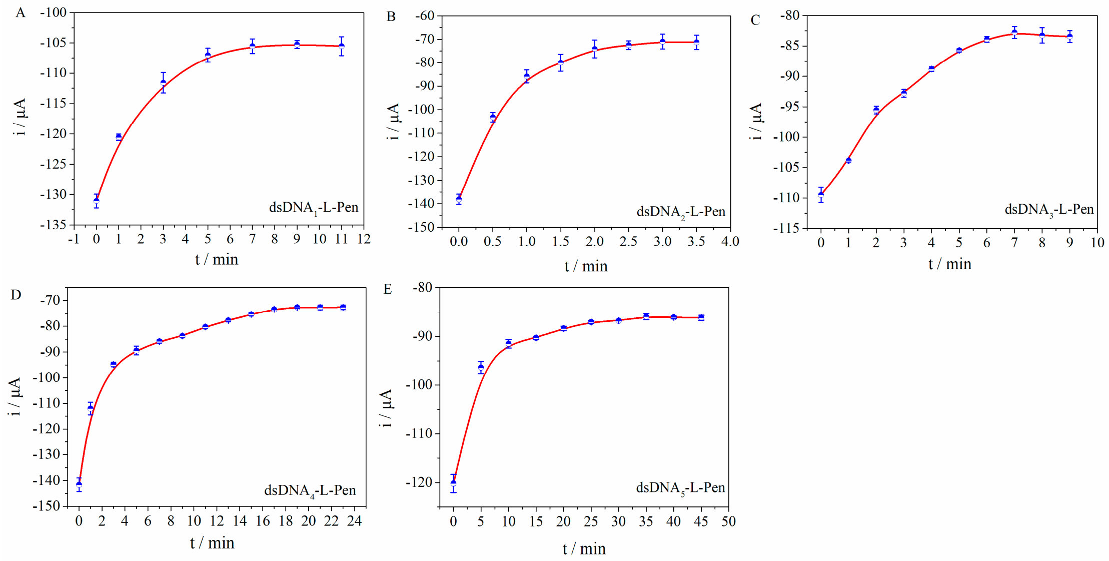

Figure S2. The effect embedding time of 1.0 × 10^{-5} mol/L of L-Pen embedded in different dsDNA in 0.005 mol/L of $K_4[Fe(CN)_6]/K_3[Fe(CN)_6]$ (contain 0.1 mol/L KCl) solution over a potential range from +0.6 V to -0.2 V, with a scan rate of 50 mV/s and pulse amplitude of 50 mV.

AGE was used to study changes in mobility and combine performance of each dsDNA and L-Pen. The results are shown in **Figure S3**, where "k" is a DNA marker with clear levels to indicate the molecular weight of DNA from top to bottom on the agarose gel; letters from "a" to "e" indicate the dsDNA structures from 1 to 5 in sequence, which had approximately the same molecular weight; and letters "f" to "j" indicate the smearing after the interaction of each dsDNA with L-Pen. After the interaction between L-Pen and $dsDNA_1$, $dsDNA_2$, and $dsDNA_3$, the molecular sizes of the three DNA structures increased and shifted slightly in AGE, indicating a weak steric hindrance toward L-Pen; therefore, the interference from analogues could be effectively eliminated. Furthermore, after the separate reactions of $dsDNA_4$ and $dsDNA_5$ with L-Pen, $dsDNA_5$ moved over a longer distance due to a smaller friction force compared to that experienced by $dsDNA_4$ in AGE: this suggests that $dsDNA_5$ was characterized by a more significant steric hindrance and difficult interaction with L-Pen. However, the combination of $dsDNA_4$ and L-Pen was relatively stable, and the internal structure matched the conformation of L-Pen, which assisted in its recognition. Therefore, the AGE experiment confirmed $dsDNA_4$ as a useful element for the auxiliary recognition of L-Pen.

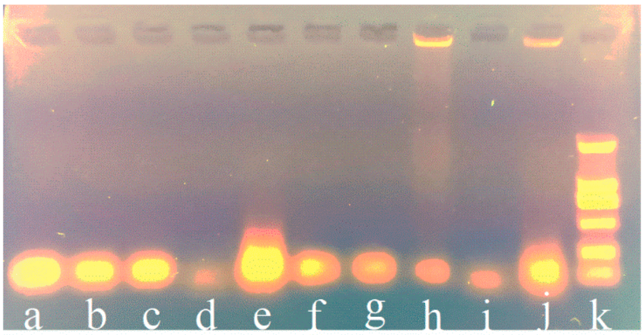

Figure S3. Characterization of L-Pen treated by each double-stranded DNA by AGE (a: $dsDNA_1$, b: $dsDNA_2$, c: $dsDNA_3$, d: $dsDNA_4$, e: $dsDNA_5$, f: $dsDNA_1$-L-Pen, g: $dsDNA_2$-L-Pen, h: $dsDNA_3$-L-Pen, i: $dsDNA_4$-L-Pen, j: $dsDNA_5$-L-Pen, k: DNA marker).

2. Electropolymerization

The thickness of the imprinted membrane affects the resorption and elution of the template molecules; therefore, it is necessary to optimize the number of polymerization cycles. As shown in **Figure S4**, the current intensity of the

oxidation peak gradually decreased with an increase in the number of scan cycles: when the number increased to 15, the oxidation peak disappeared, and the current intensity reached a plateau, indicating that a dense and weakly conductive film was formed on the surface of the dsDNA-L-Pen-modified electrode.

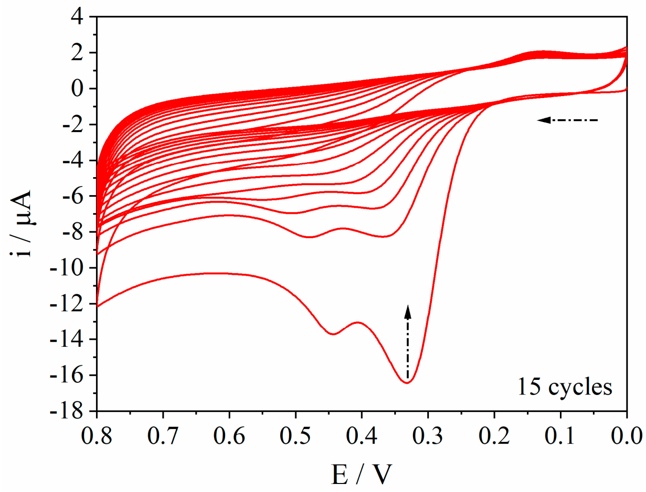

Figure S4. The formation of MIPs/dsDNA via electropolymerization (Scan rate:50 mV/s; cycle number: 15; working potential: 0 ~0.8 V).

3. The Effect of pH value on MIP/dsDNA sensor recognition of L-Pen

The pH value of eluent will affect the stability of MIP/dsDNA film and dsDNA, and then affect the elution and recognition performance. Therefore, the effect of pH value on the sensor was evaluated by the change (△*I*) in the DPV response after the eluted MIP/dsDNA sensor rebound 5.0×10^{-11} mol/L L-Pen. The removal of L-Pen from poly (*o*-phenylenediamine) membrane needs to be carried out in an alkaline environment, and excessive alkalinity will also lead to the uncoiling of dsDNA structure, and so the pH range of 7.0 to 9.0 was selected for evaluation. As shown in **Figure S5**, the rebound performance of MIP/dsDNA sensor for L-Pen was gradually enhanced with the increase of pH value of methanol-acetic acid in the eluent, and reached the maximum at pH 8.0. When the alkaline conditions of the eluate continued to increase, the rebound effect of the sensor on L-Pen was weakened, which may be due to the destruction of the stability of imprinted membrane and dsDNA and the activity of dsDNA in an over-alkaline environment, resulting in inability to orientate recognition and rebinding more L-Pen. Hence, to ensure the activity of dsDNA and the stability of imprinted membrane, pH=8.0 was chosen as the best elution environment.

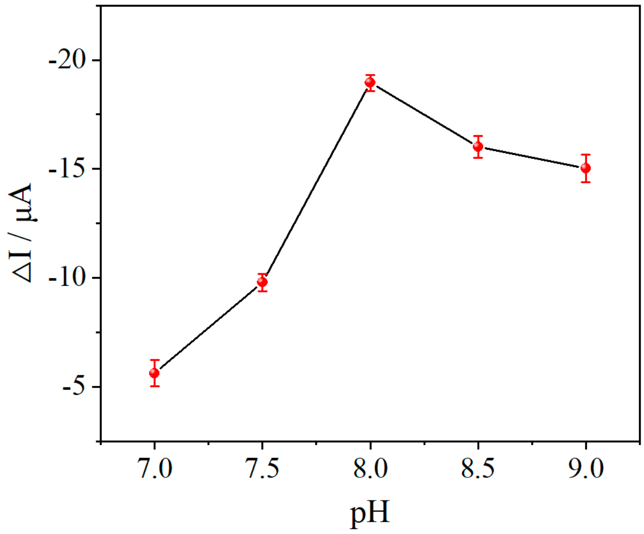

Figure S5. The Effect of eluent pH value on MIP/dsDNA sensor recognition of L-Pen.

Table S1. DNA base sequences.

Serial number	DNA sequence (ssDNA)	DNA sequence (dscDNA)
1	5'-SH-C_6-AAAAAAAAAAAA-3'	3'-C_6-TTTTTTTTTTTT-5'
2	5'-SH-C_6-ACACACACACAC-3'	3'-C_6-TGTGTGTGTGTG-5'
3	5'-SH-C_6-AGAGAGAGAGAG-3'	3'-C_6-TCTCTCTCTCTC-5'
4	5'-SH-C_6-ATATATATATAT-3'	3'-C_6-TATATATATATA-5'
5	5'-SH-C_6-CGCGCGCGCGCG-3'	3'-C_6-GCGCGCGCGCGC-5'

Table S2. Comparison of performance between this method and other methods for detecting L-Pen.

Methods	Materials	Selectivity (D-Pen/L-Pen)	Anti-interference	Linear range (mol/L)	DL (mol/L)	Ref.
Fluorescence assay	NALC-CdTe QDs	——	Anti-interference, including L-Arginine (L-Arg), L-Phenylalanine (L-Phe), L-Penicillamine (L-PA), L-Mandelic acid (L-Ma), L-histidine (L-His), L-Tyrosine (L-Tyr), L-Cysteine (L-Cys), Glutathione (GSH), L-Tryptophan (L-Try), L-Aspartic acid (L-Asp), L-Threonine (L-Thr), L-Lysine (L-Lys), L-Ascorbic acid (L-AA), and D-Tartaric acid (D-TA)	$2.0 \times 10^{-7} \sim 4.0 \times 10^{-6}$	5.80×10^{-8}	[1]
CD sensor	Cys-CdS QDs	——	Anti-interference, including L-Alanine, L-Arginine, L-Asparagine, L-Aspartic acid, L-Glutamic acid, d, L-Glutamine, L-Histidine, L-Isoleucine, L-Leucine, D,L-Methionine, D-Phenylalanine, L-Phenylalanine, L-Proline, L-Threonine, L-Tryptophan, L-Tyrosine, L-Serine and L-Valine	$1.0 \times 10^{-6} \sim 3.5 \times 10^{-5}$	7.40×10^{-7}	[2]
ECL sensor	Hb/Au-g-CN	1.52	——	$1.0 \times 10^{-4} \sim 5.0 \times 10^{-3}$	3.30×10^{-5}	[3]
Electrochemical method	BSA	——	——	$1.0 \times 10^{-8} \sim 1.0 \times 10^{-1}$	3.31×10^{-9}	[4]
Electrochemical method	DNA-MWNT	0.37	Anti-interference, including D-Pen, D-Cysteine hydrochloride, L-Cysteine hydrochloride, D-Alanine, L-Alanine and L-Valine	$1.0 \times 10^{-8} \sim 1.0 \times 10^{-1}$	3.30×10^{-9}	[5]
Electrochemical sensor	Van	1.16	——	$1.0 \times 10^{-2} \sim 1.0 \times 10^{-1}$	3.0×10^{-2}	[6]
Fluorescence	$CDs@SiO_2@IDA\text{-}Cu^{2+}$	0.40	Anti-interference, including L-Histidine, L-Aspartic Acid, L-Glutamic acid, L-Glutathione, L-Cysteine, Cl^-, Br^-, I^-, K^+, Na^+, Zn^{2+}, Pb^{2+}, and	$2.0 \times 10^{-5} \sim 1.4 \times 10^{-4}$	1.17×10^{-7}	[7]

			only D-PA has a certain selectivity			
Colorimetric sensor	CeO_2/Pal	——	10-fold anti-interference, including K^+, Ca2+, Mg^{2+}, Glu, Fru, Mal, Arg, Ser	$1.0 \times 10^{-5} \sim 1.0 \times 10^{-4}$	8.37×10^{-6}	[8]
Electrochemiluminescence	Au-BU[3]-L-Cys	——	100-fold anti-interference, including *D*-PA, K^+, Ca^{2+}, Mg^{2+}, Zn^{2+}, *L*-Try, *L*-Met, *L*-Ser, and *L*-Asn	$1.0 \times 10^{-10} \sim 1.0 \times 10^{-5}$	1.0×10^{-12}	[9]
MIPs/dsDNA sensor	dsDNA	**0.05**	1000-fold anti-interference, including L-Valine (L-Val), D-Valine (D-Val), L-Cysteine (L-Cys), D-Cysteine (D-Cys), L-Alanine (L-Ala), D-Alanine (D-Ala), and D-Pen	$3.0 \times 10^{-16} \sim 3.0 \times 10^{-13}$	2.48×10^{-16}	**This work**

— —: not found.

References

1. Huang, Y. M.; Yang, J. D.; Yuan, H. Y.; Guo, Y.; Zeng, X. Q.; Cheng, J. W.; Zhang, Y. H. A novel competitive-displacement fluorescence assay for l-penicillamine based on the reaction between the target and N-acetyl-l-cysteine-capped CdTe quantum dots for copper ions. *Anal. Methods*. **2018**, 10, 2263-2271.
2. Ngamdee, K.; Puangmali, T.; Tuntulani, T.; Ngeontae, W. Circular dichroism sensor based on cadmium sulfide quantum dots for chiral identification and detection of penicillamine. *Anal. Chim. Acta*. **2015**, 898, 93-100.
3. Lin, X.; Zhu, S.; Wang, Q. H.; Xia, Q.; Ran, P. Y.; Fu, Y. Z. Chiral recognition of penicillamine enantiomers using hemoglobin and gold nanoparticles functionalized graphite-like carbon nitride nanosheets via electrochemiluminescence. *Colloids Surf., B*. **2016**, 148, 371-376.
4. Wang, Y. H.; Han, Q.; Zhang, Q.; Huang, Y. H.; Guo, L. J.; Fu, Y. Z. Enantioselective recognition of penicillamine enantiomers on bovine serum albumin-modified glassy carbon electrode. *J. Solid State Electrochem*. **2013**, 17, 627-633.
5. Wang, Y. H.; Zhou, J.; Han, Q.; Chen, Q.; Guo, L. J.; Fu, Y. Z. Chiral Recognition of Penicillamine Enantiomers Based on DNA-MWNT Complex Modified Electrode. *Electroanalysis*. **2012**, 24, 1561-1566.
6. Wang, Y.; Han, Q.; Zhang, Q.; Huang, Y.; Guo, L.; Fu, Y. Z. Chiral recognition of penicillamine enantiomers based on a vancomycin membrane electrode. *Anal. Methods*, **2013**, 5, 5579-5583.
7. Zhang, Y.; Wang, H. Y.; He, X. W.; Li, W. Y.; Zhang, Y. K. Homochiral fluorescence responsive molecularly imprinted polymer: Highly chiral enantiomer resolution and quantitative detection of L-penicillamine. *J. Hazard. Mater.* **2021**, 412, 125249.
8. Lian, J. J.; Liu, P.; Liu, Q. Y. Nano-scale minerals in-situ supporting CeO_2 nanoparticles for off-on colorimetric detection of L–penicillamine and Cu^{2+} ion. *J. Hazard. Mater*, **2022**, 433, 128766.
9. Wu, X. F.; Ge, Q.; Jiang, N.; Liu, M.; Cong, H.; Tao, Z. Ultrasensitive sensor for L-penicillamine with chirality-induced amplification of benzo [3] uril electrochemiluminescence via supramolecular interactions. *Sens. Actuators, B* **2022**, 362, 131801-
